# Community- versus hospital-acquired acute kidney injury in hospitalised COVID-19 patients

**DOI:** 10.1186/s12882-021-02471-2

**Published:** 2021-07-23

**Authors:** Jack S Bell, Benjamin D James, Saif Al-Chalabi, Lynne Sykes, Philip A Kalra, Darren Green

**Affiliations:** 1 Salford Royal NHS Foundation Trust, Salford, UK; 2 Department of Renal Medicine, Salford Royal NHS Foundation Trust, Salford, UK; 3 Faculty of Biology, Medicine and Health, University of Manchester, Manchester, UK; 4 Emergency Assessment Unit, Salford Royal NHS Foundation Trust, Salford, UK

**Keywords:** Acute kidney injury, COVID-19, Hospital-acquired, Community-acquired, Risk factors, Mortality

## Abstract

**Background:**

Acute kidney injury (AKI) is a recognised complication of coronavirus disease 2019 (COVID-19), yet the reported incidence varies widely and the associated risk factors are poorly understood.

**Methods:**

Data was collected on all adult patients who returned a positive COVID-19 swab while hospitalised at a large UK teaching hospital between 1st March 2020 and 3rd June 2020. Patients were stratified into community- and hospital-acquired AKI based on the timing of AKI onset.

**Results:**

Out of the 448 eligible patients with COVID-19, 118 (26.3 %) recorded an AKI during their admission. Significant independent risk factors for community-acquired AKI were chronic kidney disease (CKD), diabetes, clinical frailty score and admission C-reactive protein (CRP), systolic blood pressure and respiratory rate. Similar risk factors were significant for hospital-acquired AKI including CKD and trough systolic blood pressure, peak heart rate, peak CRP and trough lymphocytes during admission. In addition, invasive mechanical ventilation was the most significant risk factor for hospital-acquired AKI (adjusted odds ratio 9.1, p < 0.0001) while atrial fibrillation conferred a protective effect (adjusted odds ratio 0.29, p < 0.0209). Mortality was significantly higher for patients who had an AKI compared to those who didn’t have an AKI (54.3 % vs. 29.4 % respectively, p < 0.0001). On Cox regression, hospital-acquired AKI was significantly associated with mortality (adjusted hazard ratio 4.64, p < 0.0001) while community-acquired AKI was not.

**Conclusions:**

AKI occurred in over a quarter of our hospitalised COVID-19 patients. Community- and hospital-acquired AKI have many shared risk factors which appear to converge on a pre-renal mechanism of injury. Hospital- but not community acquired AKI was a significant risk factor for death.

## Background

It has been more than 12 months since the first reported case of coronavirus disease 2019 (COVID-19) from Wuhan, China. As of 16th January 2021 more than 90 million confirmed cases and nearly 2 million deaths have been reported worldwide [1]. COVID-19 causes a wide spectrum of clinical manifestations ranging from asymptomatic infection to severe acute respiratory distress syndrome (ARDS) and there is recognition of a growing number of extrapulmonary complications [2]. Acute kidney injury (AKI) appears to be one of the most widely reported complications of the disease and has been associated with significantly worse outcomes [3]. However, studies to date, which originate principally from China and the United States, vary widely in their reported AKI incidence (0.5–46 %)[4–9] and risk factors for AKI remain poorly understood.

The pathophysiology of AKI in COVID-19 is presumed to be multifactorial. Histopathological case series describe acute tubular injury as the predominant finding with less frequent descriptions of thrombotic microangiopathy, cast nephropathy, and collapsing nephropathy [10–15]. There is also debate as to whether there is direct viral injury to the renal parenchyma with conflicting reports regarding the presence of SARS-CoV-2 in the proximal tubular epithelium [11–14, 16]. The clinical factors driving these pathological findings are likely a combination of traditional risk factors for AKI, such as dehydration and predisposing comorbidities, alongside risk factors which may be more specific to COVID-19 disease. Iatrogenic factors are of particular interest in COVID-19 disease due to high requirements for respiratory support, difficulties managing fluid status, and use of nephrotoxic medications. It is therefore possible that the aetiological factors for AKI in COVID-19 will differ between community- and hospital-acquired AKI.

We therefore hypothesise that the time of onset of AKI in hospitalised patients with COVID-19 will provide some differentiation of cause and therefore of risk factors and outcomes. For example, an AKI already present at the point of admission to hospital may have different pathophysiological drivers to one that occurs in a ventilated patient with a restrictive fluid management strategy. Pre-COVID-19 studies have indeed shown that risk factor profiles and outcomes are different for AKIs present on admission to hospital compared to those that develop during hospital admission [17–19]. We think this approach will be particularly informative in COVID-19 disease where there is a high propensity for iatrogenic kidney injury.

To our knowledge this is the first United Kingdom (UK) study to report on the incidence, clinical and biochemical characteristics, risk factors, and outcomes of AKI in hospitalised adult COVID-19 patients. We take the unique approach of stratifying patients into community- versus hospital-acquired AKI based on the timing of AKI onset to achieve a better understanding of relevant risk factors and outcomes.

## Methods

### Data extraction

This retrospective cohort study was conducted at Salford Royal Hospital which is a large teaching hospital in the North West of England with over 800 inpatient beds. As a global digital exemplar site, virtually all patient data is entered into an electronic patient record and anonymised patient data can be extracted via the ‘data warehouse’ for analysis. Data extraction was carried out as part of an ongoing AKI quality improvement project and therefore did not require specific ethical approval [20]. The anonymised extracted data in the AKI database contained demographics, observations, laboratory results, select medications, and comorbidities and inpatients events coded using the International Classification of Disease Tenth Revision (ICD-10). ICD coding of comorbidities was manually verified for key comorbidities (chronic kidney disease, cardiovascular disease, chronic respiratory disease, diabetes, cancer, obesity) on 45 randomly selected patients (10 % of cohort). There was 88.2 % concordance between ICD coding and manual collection. The hospital Intensive Care National Audit and Research Centre (ICNARC) reporting system was used to provide data on organ support received in critical care. A select number of further variables deemed relevant to AKI in COVID-19 disease were collected manually. These included markers of inflammation such as C-reactive protein (CRP) and lymphocyte counts [21], peak oxygen flow rate as a metric for hypoxia and severity of COVID-19 disease, and receipt of intravenous fluids.

### Participants

All adult inpatients who had a positive COVID-19 swab between 1st March 2020 and 3rd June 2020 were identified via the electronic patient record and had anonymised data extracted as detailed above. Exclusions included patients under 18 years of age, pregnant women (ICD-10 codes Z33, Z34*, Z35*), and patients with end-stage renal failure, identified via dialysis documentation within the electronic patient record or an ICD-10 code (Z94.0) indicating kidney transplant. Patients without a recorded creatinine during their stay were also excluded. In the case of patients with multiple admissions during the study period, only the first admission with a positive COVID-19 swab was considered in the analysis. Mortality included both in-hospital death and death following discharge.

### Definitions

AKIs were identified using the U.K. National Health Service AKI algorithm laboratory alert system [22] based on the Kidney Disease Improving Global Outcomes (KDIGO) AKI creatinine criteria [23]. This includes patients where historical creatinine values are unknown. Any AKI occurring 14 days before the first positive COVID-19 swab was considered to be very unlikely to be related to the COVID-19 illness and was excluded from the analysis. An AKI occurring within 48 h of admission was defined as a community-acquired AKI and an AKI occurring after 48 h was defined as a hospital-acquired AKI as described previously [18, 24].

### Statistical analysis

Proportions were presented for categorical variables, means and standard deviations for normally distributed continuous variables, and medians and interquartile ranges for skewed continuous variables. Differences between groups were compared using Fisher’s exact test for categorical variables, independent t-tests for normally distributed continuous variables, and the non-parametric Kruskal Wallis test for skewed continuous variables. All analyses were 2-tailed and a *P* value < 0.05 was considered to be statistically significant. Missing data analysis procedures used missing at random (MAR) assumptions. As the proportion of missing data was negligible (0.65 %), a complete-case approach was used in regression analyses. Statistical analyses were performed using JMP 15 (SAS institute) and SPSS software.

Stepwise regression models were used to identify independent risk factors for community- and hospital-acquired AKI. The pool of variables used in these models was divided into 3 groups:

1. Pre-admission factors: age, gender, CFS, comorbidities, pre-admission medications. Ethnicity was not included due to the low numbers of ethnic minority patients in our cohort, as detailed in the results.
2. Admission factors: admission blood results (blood results within 24 h of admission), admission observations (first set of observations taken in hospital).
3. Inpatient factors: inpatient blood results, inpatient observations, critical care admission and organ support, inpatient medications, intravenous fluids.

A mixed forward and backward stepwise regression model was used with covariates entering and exiting the model at an alpha risk level of < 0.1. This stepwise regression was first applied to pre-admission factors. Factors with an alpha risk level of < 0.1 were then pulled through to the next stage and considered alongside admission factors in a stepwise regression. For hospital-acquired AKI there was an additional stage in which factors with an alpha risk level of < 0.1 in the admission stage were considered alongside inpatient factors in a stepwise regression.

Kaplan-Meier analyses with log-rank tests were used to compare survival distributions between groups. Cox regression models were used to identify independent risk factors for death with the same stepwise procedure as described above sequentially considering pre-admission, admission and inpatient factors. AKI, invasive mechanical ventilation, peak CRP and trough lymphocytes were entered as time-dependent covariates. Worst inpatient observations were not included in this model as they could not be time-stamped and entered as time-dependent covariates.

## Results

### Baseline characteristics

**Fig. 1:**
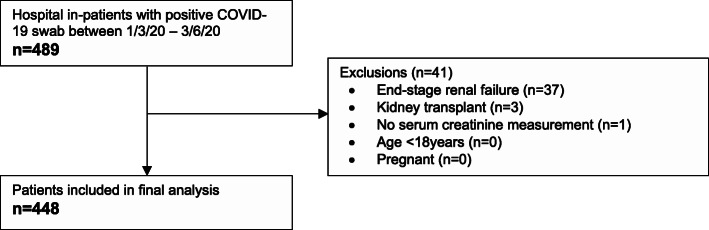
Exclusion flowchart

Between the 1st March and the 3rd June 2020, 489 patients admitted at Salford Royal Hospital returned a positive swab for COVID-19. Following exclusions for end-stage renal failure and kidney transplant, 448 patients were included in the analysis cohort (see Fig. 1). The baseline characteristics of the cohort are shown in Table 1. Of note, the mean age was 69.4 years, 54.8 % of patients were male, and 88 % were Caucasian. With regard to organ support, 13.8 % of patients were admitted to critical care, 14.1 % received non-invasive ventilation with or without invasive mechanical ventilation, and 11.6 % received invasive mechanical ventilation. 394 patients (87.9 %) had a positive COVID-19 swab within 14 days of hospital admission and were classified as community-acquired COVID-19 and 54 patients (12.1 %) had a positive COVID-19 swab after 14 days of hospital admission and were classified as hospital-acquired COVID-19.

**Table 1:** Baseline characteristics table

		Covariates	All patients (*n*=448)	No AKI (*n*=330)	Community-acquired AKI (*n*=57)	Hospital-acquired AKI (*n*=61)
Pre-admission factors	General	Male	246 (54.8)	173 (52.4)	36 (61.2)	37 (60.7)
		Age^†^	69.4 (16.2)	69.4 (16.6)	72.0 (13.9)	66.7 (15.7)
		Clinical frailty score^†^	4.2 (1.9)	4.17 (1.9)	4.8 (1.9)*	4 (1.89)
	Comorbidities	Chronic kidney disease	45 (10)	25 (7.6)	11 (19.3)*	9 (14.8)
		CV disease^a^	186 (41.4)	139 (42.1)	27 (47.4)	20 (32.8)
		Atrial fibrillation	92 (20.5)	72 (21.8)	14 (24.6)	6 (9.8)^**+**^
		Heart failure	40 (8.9)	24 (7.3)	9 (15.8)	7 (11.5)
		Hypertension	195 (43.4)	143 (43.3)	30 (52.6)	22 (36.1)
		Diabetes	117 (26.1)	76 (23.0)	22 (38.6)*	19 (31.2)
		Respiratory disease^b^	106 (23.6)	74 (22.4)	17 (29.8)	15 (24.6)
		Obesity	100 (22.3)	75 (22.7)	9 (15.8)	16 (26.2)
	Pre-admission drugs	ACEI/ARB	109 (24.3)	81 (24.6)	14 (24.6)	14 (23.0)
		Loop diuretics	68 (15.0)	48 (14.6)	9 (15.8)	11 (18.0)
Admission factors	Admission bloods	Admission CRP^‡^	79 (32, 153)	76. (29, 133)	113 (49, 210)*	85 (22, 170)
		Admission lymphocytes^‡^	0.9 (0.6, 1.3)	0.9 (0.6, 1.3)	0.8 (0.4, 1.3)	0.9 (0.7, 1.1)
		Admission urea Cr ratio^†^	94.4 (39.0)	93.9 (36.3)	97.7 (36.5)	94.2 (53.2)
	Admission observations	Heart rate^†^	91.6 (19.2)	90.7 (19.1)	95.9 (20.8)	92.3 (17.4)
		Systolic BP^†^	129.5 (22.8)	130.6 (21.6)	120.6 (29.3)*	131.5 (20.8)
		Respiratory rate^†^	22.5 (6.6)	22.1 (6.2)	24.8 (7.9)*	22.6 (7.3)
		Oxygen saturation^‡^	96 (93, 97)	96 (93, 97)	94 (93, 96)*	96 (93, 97)
		Temperature^†^	37.4 (1.1)	37.4 (1.0)	37.3 (1.3)	37.4 (1.0)
	COVID-19 acquisition	Community-acquired	394 (87.9)	294 (89.1)	57 (100)	43 (70.5)
		Hospital-acquired	54 (12.1)	36 (10.9)	0 (0)	18 (29.5)
Inpatients factors	Inpatient bloods	Peak CRP^‡^	140 (77, 239)	123 (65, 218)	196 (120, 287)	217 (116, 290)^**+**^
		Trough lymphocytes^‡^	0.6 (0.4, 0.9)	0.7 (0.5, 0.9)	0.5 (0.3, 0.8)	0.3 (0.5, 0.7)^**+**^
	Inpatient observations	Trough systolic BP^†^	99.8 (14.3)	101.9 (13.3)	93.7 (16.0)	93.0 (14.7)^**+**^
		Peak heart rate^†^	109.6 (19.2)	107.5 (18.1)	113.4 (20.0)	118.5 (21.9)^**+**^
		Peak O2 flow rate^‡^	4 (0, 13.5)	3 (0, 8)	6 (2, 15)	10 (1.3, 15)^**+**^
	Inpatient drugs	ACEI/ARB	60 (13.3)	52 (15.8)	3 (5.3)	5 (8.2)
		Loop diuretics	104 (23.2)	66 (20)	12 (21.1)	26 (42.6)
		IV fluids on admission	269 (60.0)	189 (57.3)	46 (80.7)	34 (55.7)
	Organ support	Critical care admission	62 (13.8)	29 (8.8)	11 (19.3)	22 (36.1)^**+**^
		IMV	52 (11.6)	19 (5.8)	10 (17.5)	23 (37.7)^**+**^
		NIV alone	27 (6.0)	20 (6.1)	4 (7.0)	3 (4.9)
		Vasopressors	64 (14.3)	32 (9.7)	10 (17.5)	22 (36.1)^**+**^
		RRT	14 (3.1)	1 (0.3)	3 (5.3)	10 (16.4)^**+**^
	Peak AKI stage	AKI stage 1	65 (14.5)	N/A	28 (49.1)	37 (60.7)
		AKI stage 2	22 (4.9)	N/A	13 (22.8)	9 (14.8)
		AKI stage 3	31 (6.9)	N/A	16 (28.1)	15 (24.6)
	Outcomes	Still an inpatient	40 (8.9)	28 (8.5)	1 (1.8)	11 (18.0)
		Discharged	275 (61.4)	225 (68.2)	31 (54.4)	19 (31.2)^**+**^
		Died	161 (35.9)	97 (29.4)	30 (52.6)*	34 (55.7)^**+**^
		Length of stay in days^‡^	7 (4, 14)	7 (3, 13)	7 (3.8, 13)	16.5 (7.3, 33)^**+**^

Values are presented as n (proportion) unless specified in the covariate columns:^†^indicates values presented as mean (standard deviation) and^‡^indicates values presented as median (interquartile range).

*Denotes a significant difference (*p*<0.05) when comparing AKI present on admission (community-acquired AKI) to no AKI present on admission to hospital by univariate analysis.

^**+**^Denotes a significance difference (*p*<0.05) when comparing hospital-acquired AKI to no AKI during admission by univariate analysis.

^a^Cardiovascular disease includes ischaemic heart disease, stroke, peripheral vascular disease, valvular heart disease, atrial fibrillation and heart failure.

^b^Respiratory disease includes asthma, COPD, pulmonary fibrosis and bronchiectasis.

Abbreviations:*CV *cardiovascular;*ACEI* angiotensin converting enzyme inhibitor;*ARB *angiotensin II receptor blocker;*CRP *C-reactive protein;*BP *blood pressure;*IMV *invasive mechanical ventilation;*NIV *non-invasive ventilation;*RRT *renal replacement therapy;*Cr *creatinine

### Incidence of AKI

**Fig. 2:**
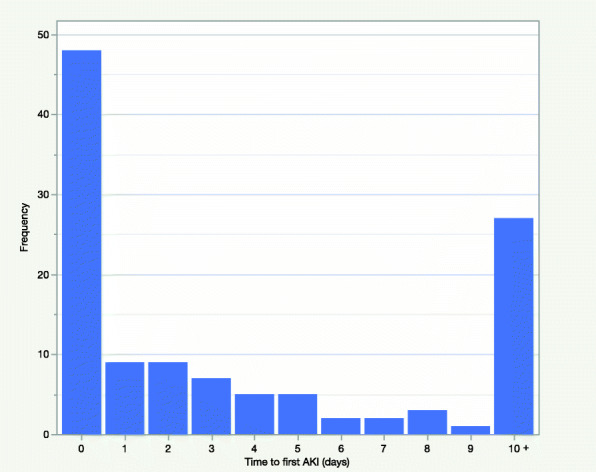
Time to first AKI from admission to hospital

A total of 118 out of 448 patients (26.3 %) recorded an AKI during their admission. AKI incidence was higher in critical care where 33 out of 62 patients (53.2 %) recorded an AKI. The proportion of AKI patients with a peak AKI stage of 1, 2 and 3 was 55.1 %, 18.6 %, and 26.3 % respectively. 11.9 % of AKI patients received renal replacement therapy. AKI patients were separated into 2 groups based on the timing of AKI onset relative to hospital admission: 57 patients had AKI onset within 48 h of hospital admission and were categorised as community-acquired AKI, and 61 patients had AKI onset after 48 h of hospital admission and were categorised as hospital-acquired AKI. Figure 2 shows the timing of AKI onset relative to admission to hospital.

### Risk factors for community-acquired AKI

A stepwise regression model was used to identify independent risk factors for community-acquired AKI. When considering pre-admission factors in a multivariable model, only clinical frailty score and CKD were significant independent risk factors for community-acquired AKI while diabetes was of borderline significance (Table 2). Age, other major comorbidities and pre-admission use of loop diuretics and drugs that act on the renin-angiotensin-aldosterone system were not significant in this model.

**Table 2:** Independent risk factors for community-acquired acute kidney injury by multivariable analysis

**Covariates**	**Adjusted odds ratios**	**95% confidence intervals**	***P*value**	
Stage 1: Pre-admission factors only				
Clinical frailty score	1.18	1.01 - 1.38	0.0333	
CKD	2.18	1.02 - 4.7	0.0448	
Diabetes	1.78	0.98 - 3.23	0.0597	
Stage 2: Pre-admission and admission factors				
Clinical frailty score	1.19	1.01 - 1.41		0.0389
CKD	2.36	1.06 - 5.26		0.0359
Diabetes	2.15	1.14 - 4.05		0.0177
Admission CRP per 10 units	1.04	1.01 - 1.07		0.0174
Admission systolic blood pressure	0.98	0.96 - 0.995		0.0085
Admission respiratory rate	1.05	1.01 - 1.10		0.0131

Abbreviations: *CKD* chronic kidney disease; *CRP* C-reactive protein

When admission observations and blood results were included in the stepwise regression alongside the pre-admission factors that reached an alpha risk level of < 0.1, lower systolic blood pressure, higher respiratory rate, and higher admission CRP became the most significant independent risk factors for community-acquired AKI. In this model, clinical frailty score, CKD and diabetes were also significant (Table 2).

### Risk factors for hospital-acquired AKI

A similar stepwise approach was used to identify risk factors for hospital-acquired AKI by sequentially considering pre-admission factors, admission factors and inpatient factors. Factors with an alpha level of risk < 0.1 were pulled through to the next stage of the model. Considering pre-admission factors alone, atrial fibrillation (AF) and CKD were significant risk factors for hospital-acquired AKI, with AF conferring a protective effect (adjusted odds ratio 0.36) (Table 3). Addition of admission factors such as admission observations and blood results to the stepwise regression identified no new significant factors (Table 3). When inpatient factors were added into the model, invasive mechanical ventilation, trough systolic blood pressure during spell, peak heart rate during spell, peak CRP and trough lymphocytes became significant independent risk factors for AKI alongside CKD and AF (Table 3). Of note, AF remained a significant independent protective risk factor for hospital-acquired AKI after controlling for peak heart rate. Of the AF patients, 78 % had pre-existing AF and 22 % had new onset AF during admission. 82 % of the patients with pre-existing AF were anticoagulated prior to admission.

**Table 3:** Independent risk factors for hospital-acquired AKI by multivariable analysis

**Covariates**	**Adjusted odds ratios**	**95% confidence intervals**	***P*value**	
Stages 1 and 2: Pre-admission and admission factors				
CKD	2.42	1.05 - 5.58	0.0388	
Atrial fibrillation	0.36	0.15 - 0.88	0.0244	
Stage 3: Pre-admission, admission factors and inpatient factors				
CKD	3.65	1.24 - 10.75		0.0191
Atrial fibrillation	0.29	0.10 - 0.83		0.0209
Mechanical ventilation	9.10	3.63 - 22.80		<0.0001
Trough systolic blood pressure	0.95	0.93 - 0.98		0.0001
Peak heart rate	1.03	1.01 - 1.05		0.0013
Peak CRP per 10 units	1.04	1.00 - 1.07		0.0252
Trough lymphocytes	0.28	0.08 - 0.96		0.0424

Abbreviations: *CKD* chronic kidney disease; *CRP* C-reactive protein

**Fig. 3:**
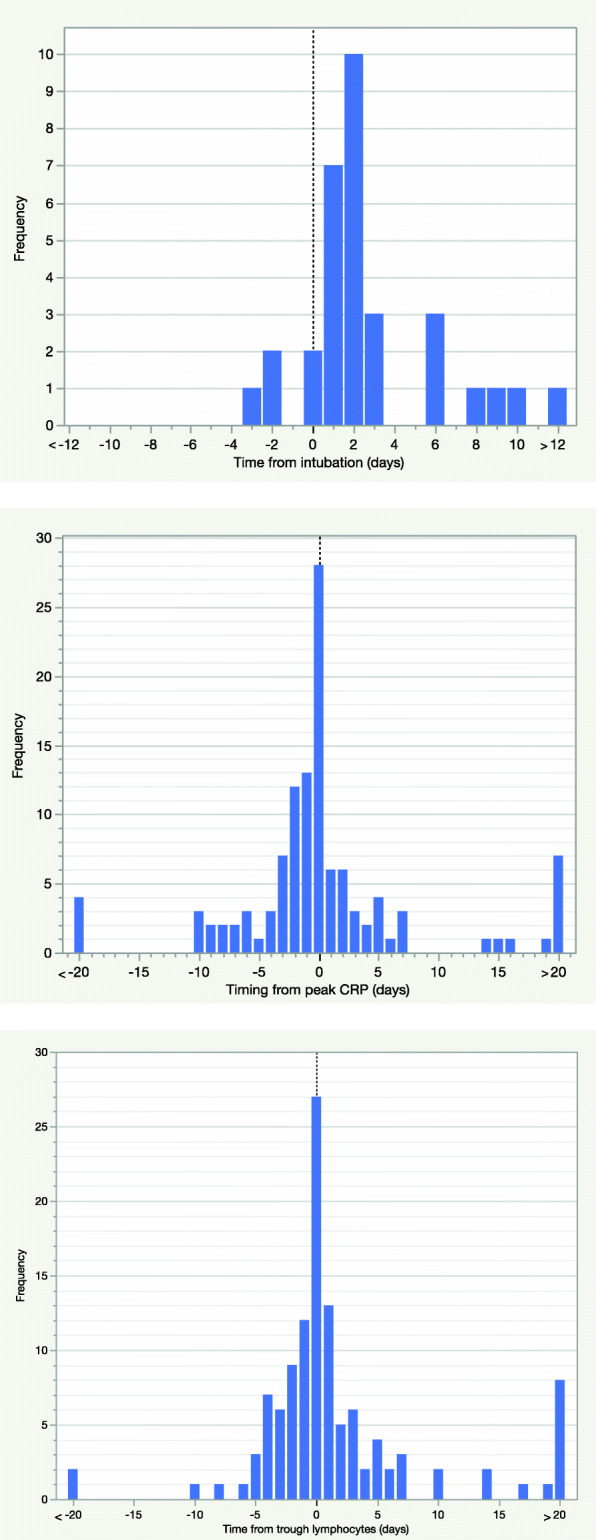
**a** Timing of AKI onset relative to timing of intubation. **b** Timing of AKI onset relative to timing of peak CRP. **c **Timing of AKI onset relative to timing of trough lymphocytes

With regard to invasive mechanical ventilation, there was significantly higher incidence of AKI (66.7 % vs. 21.5 %; p < 0.0001), AKI 3 (31.3 % vs. 4 %; p < 0.0001), and renal replacement therapy (10 % vs. 1 %; p < 0.0001) in patients who received invasive mechanical ventilation compared with those who did not. The median time from intubation to first AKI was 2 days (IQR 1–3) (Fig. 3a) with 62.5 % of AKIs occurring from day 2 of invasive mechanical ventilation onwards. There was also a strong temporal relationship between the onset of AKI and the timing of peak CRP (median time from peak CRP to AKI onset 0 days, IQR − 2–2; Fig. 3b) and trough lymphocytes (median time from trough lymphocytes to AKI onset 0 days, IQR − 3–2; Fig. 3c).

### Mortality according to AKI status

**Fig. 4:**
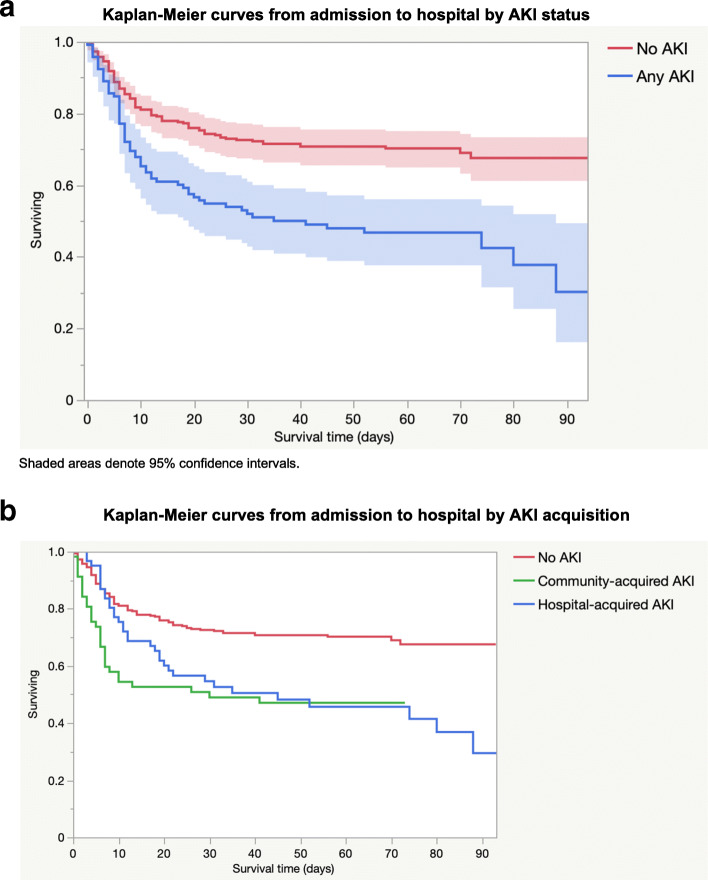
**a** Kaplan-Meier curves from admission to hospital by AKI status. **b** Kaplan-Meier curves from admission to hospital by AKI acquisition

The overall proportion of patients who died in the cohort was 35.9 %. A significantly higher proportion of patients who had an AKI died (54.3 %) compared to patients who didn’t have an AKI (29.4 %) (p < 0.0001) and the survival distributions were significantly different between patients with and without AKI (logrank p < 0.0001) (Fig. 4a). The proportion of patients who died also increased with AKI stage: for the no AKI, AKI stage 1, AKI stage 2, and AKI stage 3 groups the proportion of patients who died was 29 %, 48 %, 50 and 71 % respectively. The proportion of patients with community- and hospital-acquired AKI who died was 52.6 and 55.7 % respectively and there was no significant difference between the survival distributions of community- and hospital-acquired AKI (logrank p = 0.4345) (Fig. 4b). The median time to death after AKI onset was 5 days (IQR 2–9.75).

### Independent risk factors for death in COVID-19 patients

A stepwise Cox regression model was used to identify independent risk factors for death in COVID-19 patients and to ascertain whether community- and hospital-acquired AKI were among these. As described earlier pre-admission, admission, and inpatient factors were considered sequentially with factors meeting an alpha risk level < 0.1 being pulled through to the next stage of the model. Only inpatient variables that had timings associated with them were included in the model and were coded as time-dependent covariates. Considering pre-admission factors alone, age, clinical frailty score and obesity were significant risk factors for death (Table 4). When admission factors including community-acquired AKI were included in the stepwise regression, admission oxygen saturations, respiratory rate and CRP became significant while obesity fell out of the model (Table 4). Of note community-acquired AKI was not a significant risk factor for death when controlling for these factors. When inpatient factors including hospital-acquired AKI were then included in the stepwise regression, hospital-acquired AKI, peak CRP and trough lymphocytes became significant risk factors for death while admission CRP dropped out of the model (Table 4). Hospital-acquired AKI had one of the strongest effects on the model with a hazard ratio of 4.63 and a *p* value of < 0.0001.

**Table 4:** Independent risk factors for death using a stepwise Cox regression model

**Covariates**	**Adjusted hazard ratio**	**95% confidence intervals**	***P*value**
Stage 1: Pre-admission factors			
Age (in decades)	1.37	1.20 – 1.56	<0.0001
Clinical frailty score	1.18	1.07 – 1.31	0.002
Obesity	1.57	1.05 – 2.34	0.029
Stage 2: Pre-admission and admission factors (including community-acquired AKI)			
Age (in decades)	1.33	1.16 – 1.53	<0.0001
Clinical frailty score	1.24	1.12 – 1.38	<0.0001
Admission O2 saturation	0.95	0.92 – 0.97	<0.0001
Admission respiratory rate	1.06	1.03 – 1.08	<0.0001
Admission CRP (per 10 units)	1.03	1.01 – 1.04	0.005
Stage 3: Pre-admission, admission and inpatient factors (including hospital-acquired AKI)			
Age (in decades)	1.35	1.18 – 1.55	<0.0001
Clinical frailty score	1.26	1.13 – 1.41	<0.0001
Admission O2 saturation	0.95	0.93 – 0.98	<0.0001
Admission respiratory rate	1.04	1.02 – 1.07	<0.0001
Hospital-acquired AKI	4.64	2.98 – 7.23	<0.0001
Peak CRP (per 10 units)	1.05	1.03 - 1.06	<0.0001
Trough lymphocytes	1.14	1.03 – 1.26	0.015

Abbreviations: *AKI* acute kidney injury; *CRP* C-reactive protein

### Other relevant outcomes

By univariate analysis, hospital-acquired AKI was significantly associated with critical care admission (*p* < 0.0001), requirement for invasive mechanical ventilation (*p* < 0.0001), requirement for renal replacement therapy (*p* < 0.0001), longer length of stay (*p* < 0.0001), and reduced likelihood of discharge (*p* < 0.0001)(Table 1). Community-acquired AKI, however, was not significantly associated with any of these outcomes.

## Discussion

This is the first UK study to report on the incidence, risk factors and outcomes of AKI in COVID-19 patients. Further, by stratifying patients into community- and hospital-acquired AKI we show differences in the risk factors and outcomes of these two subgroups.

The incidence of AKI in our cohort was 26.3 % which sits broadly in-between figures reported in the US (36.6–46 %)[4, 5] and China (0.5–9.4 %)[6–8]. While it is not entirely clear why there is such a large variation in AKI incidence, it may be related to differences in the burden of comorbid disease, threshold for hospitalisation or respiratory support, racial diversity, and baseline incidences of AKI between countries. Comparing the characteristics of the largest US[4], UK (this study) and Chinese [6] COVID-19 AKI cohorts reveals differences in the proportion of males (61 % vs. 54 % vs. 52 %), the rates of diabetes (33 % vs. 26 % vs. 14 %) and hypertension (56 % vs. 43 % vs. 33 %), and requirement for invasive mechanical ventilation (22 % vs. 12 % vs. 13 %). With regard to race, our UK cohort comprised 88 % Caucasian patients while the US cohort comprised 39 % Caucasian and 20.6 % black patients, with black race subsequently being identified as a risk factor for AKI [4].

For community-acquired AKI, the most significant pre-admission risk factors were clinical frailty score, CKD and diabetes. This highlights high-risk patient groups who may benefit from increased screening for AKI in the community if COVID-19 disease is suspected. When admission factors were included in the model the most significant risk factors for community-acquired AKI became those related to disease severity and systemic upset on admission to hospital: lower systolic blood pressure, higher respiratory rate and higher CRP. These factors most likely converge on a pre-renal mechanism with hypotension, hypoxia and a systemic inflammatory response acting on a background of well-known pre-admission risk factors for AKI, such as CKD and diabetes.

For hospital-acquired AKI, the significant pre-admission risk factors were CKD and AF, with AF unexpectedly conferring a protective effect against AKI. One plausible mechanism for this may relate to the influence of therapeutic anticoagulation on the well-documented coagulopathy seen in COVID-19 [25]. Therapeutic anticoagulation could influence AKI directly by reducing renal thrombotic disease and/or indirectly by reducing pulmonary thrombotic disease, and therefore hypoxia and need for respiratory support. Renal thrombotic microangiopathy has been observed only infrequently in histopathological studies to date [11–15] but thrombotic disease in the lungs is better recognised [25]. The dosage of prophylactic anticoagulation in COVID-19 is still to be determined with some expert panels recommending routine prophylactic-dose anticoagulation for all patients without a contra-indication [26] while others suggest an increased dose for high risk patients particularly in the critical care setting [27, 28].

When inpatient factors were included in the model invasive mechanical ventilation became the most significant risk factor for hospital-acquired AKI. Further, in patients receiving invasive mechanical ventilation the median time to AKI onset was day 2 following intubation which suggests a temporal link. Invasive mechanical ventilation may increase the risk of AKI firstly by reflecting the extent of respiratory failure and systemic illness, secondly through the associated aim for neutral or negative fluid balance, and thirdly through the haemodynamic effects of positive pressure ventilation, namely reduced cardiac preload and secondary neurohumoral changes [29, 30]. Indeed, pre-COVID-19 studies have identified both invasive mechanical ventilation and ARDS as significant and independent risk factors for AKI in the critically ill [31].

Markers of haemodynamic instability (trough systolic blood pressure and peak heart rate), and markers of inflammation (peak CRP and trough lymphocytes) were also significant in-patient risk factors for hospital-acquired AKI. Haemodynamic instability is one of the few risk factors we have identified that can be modified and so careful attention should be paid to optimising fluid status. Peak CRP and trough lymphocytes have previously been shown to be significant risk factors for AKI in COVID-19 [21], and had a strong temporal relationship with the onset of AKI in our cohort. Interestingly, CRP and lymphocytes remained independent risk factors for AKI after controlling for trough systolic blood pressure and peak heart rate which raises the possibility that inflammatory mediators may cause a direct, as well as indirect, insult to the kidneys. With regard to notable absences on multivariable analysis, age, gender and ACE inhibitor/angiotensin receptor blocker use had no significant or borderline significant influence on the risk of AKI, contrary to previous studies [4, 32].

AKI was associated with significantly increased mortality in COVID-19 patients with higher AKI stage portending a worse prognosis. However, on Cox regression only hospital-acquired AKI had a significant effect on mortality with an adjusted odds ratio of 4.6, while community-acquired AKI did not. The community- and hospital-acquired AKI subgroups were compared to try to explain this difference. Firstly, it is important to note that hospital-acquired AKI was not associated with more severe AKI (peak AKI stage 2 or 3) when compared to community-acquired AKI (39.3 % vs. 50.9 % respectively). Secondly, 30 % of the hospital-acquired AKI group were categorised as hospital-acquired COVID-19 (the first positive COVID-19 swab occurred after 14 days of hospital admission) as opposed to 0 % in the community-acquired AKI group. It could be argued that patients who acquire COVID-19 while hospitalised for a different presenting complaint may be more comorbid or deconditioned than patients who are admitted to hospital with community-acquired COVID-19. However, rates of AKI and death were not significantly different between community- and hospital-acquired COVID-19 patients. Thirdly, hospital-acquired AKI patients were generally younger (age 66.7 vs. 72) and less frail (clinical frailty score 4 vs. 4.8) but were more likely to receive invasive mechanical ventilation (37.7 % vs. 17.5 %), vasopressors (36.1 % vs. 17.5 %), and loop diuretics (42.6 vs. 21.2 %) than community-acquired AKI patients. This suggests that overall disease severity may be higher in hospital-acquired AKI patients and that the iatrogenic factors associated with critical care admission may be in part be responsible for this difference in mortality. However, none of these factors were significant in the multivariable Cox regression. Fourthly, one must consider whether there are differences in the provenance of community- and hospital-acquired AKI that leads to a poorer outcome in hospital-acquired AKI patients, with iatrogenic and thrombotic disease being potential additional mechanisms in hospital-acquired AKI. Finally, pre-COVID-19 studies have shown that hospital-acquired AKI has significantly higher mortality than community-acquired AKI [18, 19]. One suggested explanation was that all patients receive senior review on admission while senior review may be more sporadic during the inpatient stay. This may lead to better recognition and management of community-acquired AKI compared to hospital-acquired AKI.

The strengths of this study lie in the stratification of AKI patients into community- and hospital-acquired AKI. Firstly, this helps us identify risk factors that are more relevant to the onset of AKI. Secondly, community- and hospital-acquired AKI are known to be associated with different risk factor profiles [17] and outcomes [18, 19]. Another strength of our study is the use of a staged model which considers pre-admission, admission and inpatient factors sequentially. This allows identification of relevant risk factors at each stage of admission.

This study also has certain limitations. Firstly, we relied on accurate inputting of data into the electronic patient record and subsequent ICD-10 coding. However, much effort was put into manually verifying and cleaning the data. Secondly, the cohort was predominantly Caucasian which limits the generalisability of our findings. Thirdly, a baseline creatinine was not available for all patients which may lead to underreporting of AKI via the laboratory AKI algorithm. Fourthly, data on radiological investigations were not readily extractable from the electronic patient record and were therefore not used as a variable in our analyses.

## Conclusions

We found that AKI occurred in over a quarter of hospitalised COVID-19 patients. The majority of risk factors for community- and hospital-acquired AKI are shared and point to a predominantly pre-renal mechanism of injury, in concordance with pathological studies to date. In addition, hospital-acquired AKI was significantly associated with invasive mechanical ventilation and AF, pointing to potential iatrogenic and thrombotic mechanisms relevant only for hospital-acquired AKI. Importantly, hospital- but not community-acquired AKI was a significant risk factor for death in COVID-19. Further work is certainly required to tease out the pathophysiology of AKI in COVID-19 patients, particularly with regard to thrombotic disease, and to determine whether this varies between community- and hospital-acquired AKI. In the meantime, the prognostic significance of hospital-acquired AKI should be recognised and acted upon promptly by managing key modifiable risk factors such as fluid status.

## Data Availability

The datasets used during the current study are available from the corresponding author on reasonable request.
